# Genotoxicity Induced by Foetal and Infant Exposure to Magnetic Fields and Modulation of Ionising Radiation Effects

**DOI:** 10.1371/journal.pone.0142259

**Published:** 2015-11-11

**Authors:** Ion Udroiu, Antonio Antoccia, Caterina Tanzarella, Livio Giuliani, Francesca Pacchierotti, Eugenia Cordelli, Patrizia Eleuteri, Paola Villani, Antonella Sgura

**Affiliations:** 1 Dept. of Science, University of Rome “Roma Tre”, Rome, Italy; 2 Research Center of Monteporzio Catone, INAIL, Rome, Italy; 3 Technical Unit for Radiation Biology and Human Health, ENEA, Rome, Italy; National Research Council, ITALY

## Abstract

**Background:**

Few studies have investigated the toxicity and genotoxicity of extremely low frequency magnetic fields (ELF-MF) during prenatal and neonatal development. These phases of life are characterized by cell proliferation and differentiation, which might make them sensitive to environmental stressors. Although *in vitro* evidences suggest that ELF-MF may modify the effects of ionizing radiation, no research has been conducted so far *in vivo* on the genotoxic effects of ELF-MF combined with X-rays.

**Aim and methods:**

Aim of this study was to investigate in somatic and germ cells the effects of chronic ELF-MF exposure from mid gestation until weaning, and any possible modulation produced by ELF-MF exposure on ionizing radiation-induced damage. Mice were exposed to 50 Hz, 65 μT magnetic field, 24 hours/day, for a total of 30 days, starting from 12 days post-conception. Another group was irradiated with 1 Gy X-rays immediately before ELF-MF exposure, other groups were only X-irradiated or sham-exposed. Micronucleus test on blood erythrocytes was performed at multiple times from 1 to 140 days after birth. Additionally, 42 days after birth, genotoxic and cytotoxic effects on male germ cells were assessed by comet assay and flow cytometric analysis.

**Results:**

ELF-MF exposure had no teratogenic effect and did not affect survival, growth and development. The micronucleus test indicated that ELF-MF induced a slight genotoxic damage only after the maximum exposure time and that this effect faded away in the months following the end of exposure. ELF-MF had no effects on ionizing radiation (IR)-induced genotoxicity in erythrocytes. Differently, ELF–MF appeared to modulate the response of male germ cells to X-rays with an impact on proliferation/differentiation processes. These results point to the importance of tissue specificity and development on the impact of ELF-MF on the early stages of life and indicate the need of further research on the molecular mechanisms underlying ELF-MF biological effects.

## Introduction

The possible increased risk of cancer–especially childhood leukaemia–related with extremely low frequency magnetic fields (ELF-MF) is cause of concern [1,2]. Many epidemiological studies have been published, but a clear association between exposure to ELF-MF and cancer has not been unequivocally demonstrated [3]. Although IARC has classified this physical agent as “possibly carcinogenic to humans (group 2B)” [4], the over 1,000 mechanistic studies conducted so far have not yet revealed the possible biologic mechanism by which ELF-MF can cause any health effect [5].

Since DNA damage is considered to be the primary cause of cancer, many studies investigated the ability of ELF-MF to harm the genome. These comprise a large number of investigations, both *in vitro* and *in vivo*, but the results are still contradictory (as reviewed in [6]). Among genotoxicity assays, the micronucleus test is one of the most used, because of its simplicity, sensitivity and reliability. This test plays an important role due to its good predictivity for carcinogenic hazards [7]. So far, there are few reports on *in vivo* genotoxic effects of ELF-MF using micronucleus assays. Svedenstal and Johanson [8] detected no differences in micronucleated erythrocytes between adult mice exposed for 90 days to a 14 μT magnetic field and those unexposed; the same result was observed by Abramsson-Zetterberg and Grawé [9], using an equal field, both in adult and newborn mice. Conversely, positive results were found analyzing erythrocytes of newborn mice prenatally exposed to 650 μT [10, 11], adult rats exposed to 1 mT for 45 days [12], adult mice exposed to 5 μT for 40 days [13] and adult mice exposed to 200 μT for 7 days [14]. Since one of the major causes of concern regarding non-ionizing radiation is their possible association with childhood cancer, it would be meaningful to study ELF-MF effects during infancy. Moreover, studying the effects induced by ELF-MF in foetal and neonatal life stages may be useful to disclose their genotoxic properties, because infant cells [15–18] and even more fetal cells [19] showed a greater sensitivity to genotoxic insults than adult cells. To our knowledge, only two works investigated genotoxic effects of ELF-MF in rodents exposed *in utero*, giving opposite results [9,10]. The importance to investigate *in utero* exposure for assessing potential carcinogenicity of ELF-MF has also been pointed out [20].

Moreover, foetal life is a critical step also in the development of male reproductive system. In rodents, primordial germ cells alternate between mitotic activity and quiescence and, differently from adults, also Sertoli cells proliferate actively [21]. There are some evidences that ELF-MF exposure might affect male reproductive system in the adult [22,23], although other studies reported negative results [24]. Few studies exist on foetal exposure to ELF-MF of male reproductive system. Results of a multigeneration study in rats did not support the hypothesis of a reproductive or developmental toxic effect [25], and no alterations in the offspring spermatogenesis and fertility were observed in rats after *in utero* and neonatal exposure [26]. On the other hand, McGivern and coworkers [27] observed an increase of weight in epididymus, prostate and seminal vesicles in adult rats exposed *in utero*. An alteration of spermatogenic epithelium was also observed in adult rats after *in utero* exposure [28] and after *in utero*/neonatal exposure [29].

Although there is some evidence that ELF-MF may interfere with the DNA damage response elicited by IR *in vitro* [30–32], no study has been conducted *in vivo* on the possible genotoxic effects of a combined exposure to ELF-MF and X-rays.

Hence, in this work we aimed at studying the effects of low-level, chronic ELF-MF exposure in mouse during a very sensitive period such as the foetal and neonatal life and any possible modulation that ELF-MF exposure might exert on damage induced by IR. Furthermore, since it has long been demonstrated [33] that IR can produce delayed effects (*de novo* effects in the unirradiated descendants or neighbours of irradiated cells), but there are no data on the influence that combined exposures to multiple physical agents may have on delayed effects, we also investigated the outcomes of combined exposures on genomic instability.

## Materials and Methods

### Animals and exposure system

Pregnant CD-1 Swiss (outbred) mice (Charles River, Italy) were divided into four groups, comprising two dams each. One group was unexposed and served as control (C, 27 pups); another group (E, 20 pups) was exposed to ELF-MF from day 11.5 post conception (p.c.) until weaning, for a total of 30 days; another group (X, 25 pups) was X-irradiated [1 Gy) on day 11.5 p.c.; the last group (XE, 31 pups) was X-irradiated (1 Gy) on day 11.5 p.c. and immediately exposed to ELF magnetic fields until weaning (30 days in total).

For this experiment, we employed a magnetic field of 65 μT. Since mice require a 12-26-fold greater MF exposure than that required by humans to induce similar current density within the body [34], the field we used is (in term of biological effects) comparable to a 2–5 μT MF for humans. These values are usually present in most households.

The 50 Hz, 65 μT magnetic field was generated by a solenoid working 24 h per day. The solenoid was 0.8 m in length and 0.13 m in radius, with 552 turns of 2.5 mm^2^ copper wire, wound in two layers in continuous forward-backward fashion around a cylinder of PVC. It was supplied by 50 Hz main power through a transformer. A voltage of 6.5 V (rms) was applied to obtain a flux density of 65 μT (rms) at the centre of the solenoid. The field was uniform between ±5% in the volume where the mice were exposed. The solenoid was not shielded for the electric field, as the induced electric field was negligible due to the low voltage used. Exposure to 1 Gy X-rays was performed in a Gilardoni apparatus (Gilardoni, Italy) at a dose rate of 0.5 Gy/min.

Animals were housed in polycarbonate cages put inside an operating solenoid (groups E and XE) or a switched off solenoid (groups C and X). The temperature and the relative humidity of the animal room were 22°C and 40%, respectively. Artificial lighting was from 8 am to 8 pm and commercial pellets and tap water were available ad libitum throughout the experimental period. The temperature inside the coils was the same as in the room.

Litter size was determined at birth. Pups were weighed at delivery and at day 11, 21, 42 and 140 after birth. Survival rate was assessed at weaning (day 21). Pups were daily monitored for appearance of physiological landmarks of development.

Forty-three male mice were sacrificed at 42 days after birth for the analyses on the reproductive system. The remaining animals were sacrificed at day 140 after birth. Sacrifice was performed by cervical dislocation in anesthetized animals.

The experiment was conducted according to Italian laws regulating the use and humane treatment of animals for scientific purposes (decree n. 116/1992). All experimental procedures were approved by the Animal Research Ethical Committee of the Italian Ministry of Health (approval ID: 10.10.15).

### Micronucleus test on erythrocytes

Blood smears were obtained puncturing the tail vein of the mice at birth and on day 11 (infancy), 21 (weaning), 42 (sexual maturity) and 140 (adulthood) after birth. The slides were fixed in absolute methanol and maintained at -20°C until staining. Smears were stained with acridine orange (Sigma-Aldrich, Italy, 20 μg/mL in pH 6.8 Sørensen buffer). The samples were coded and scored blindly by the same analyst. Micronuclei were scored at 1000× magnification using a Zeiss Axiophot (Zeiss, Germany) fluorescence microscope (494 nm excitation filter, 523 nm barrier filter). For each animal, 2,000 erythrocytes were analyzed [10].

### Comet assay on epididymal sperm

After sacrifice, both epididymal caudae were surgically removed, placed in a Petri dish containing TNE buffer (0.15 M NaCl, 0.01 M Tris-HCl, 0.001 M Na_2_EDTA, pH 7.4) and minced with curved scissors. Sperm suspensions were filtered through a 0.2 mm nylon mesh, centrifuged (5 min, 1800 g), resuspended in TNE buffer plus 10% glycerol, aliquoted, and frozen at -80°C. Alkaline (pH 12.1) and neutral (pH 8.0) protocols of Comet assay were performed to evaluate DNA damage in spermatozoa. The assay was performed essentially according to Cordelli et al. [35]. Briefly the slides were immersed in a lysing solution (2.5 M NaCl, 100 mM Na_2_EDTA, 10 mM Tris, pH 10.0) containing 10% DMSO (Carlo Erba, Italy) and 1% Triton X-100 (Sigma-Aldrich), overnight at 4°C. At the end of lysis slides were immersed for 30 minutes in 10 mM dithiothreitol (Sigma-Aldrich) in lysis solution to decondense the extremely compacted sperm chromatin and allow the migration of DNA. Slides were then placed in a horizontal gel electrophoresis tank where electrophoresis was performed under the following conditions: 10 minutes at 4°C in alkaline electrophoresis buffer (300 mM NaOH, 1 mM Na_2_EDTA; HCl was added to reach pH 12.1), followed by 7 minutes of 27 V (0.8 V/cm), 300 mA electrophoresis, at 4°C, for the alkaline assay; 20 minutes in TBE buffer (2 mM Na_2_EDTA, 90 mM Tris, 90 mM boric acid; pH 8.0) at 4°C, followed by 7 minutes of 27 V (0.8 V/cm), 10 mA electrophoresis, at 4°C, for the neutral assay. After electrophoresis, the slides were fixed for 5 minutes in Tris 0.4 M pH 7.5, and for 5 minutes in absolute ethanol and were air-dried at room temperature. Immediately before scoring, slides were stained with 12 μg/mL ethidium bromide (Sigma-Aldrich) and examined at 200× magnification with an Olympus fluorescence microscope. Slides were analyzed blindly with a computerized image analysis system (Delta Sistemi, Italy). To evaluate the amount of DNA damage, 100 cells were analysed from two different slides, and computer generated percentage of tail DNA values (tail intensity) were used.

### Flow cytometric analysis of testicular cell subpopulations distribution

After removal of the tunica albuginea, the testes were minced with surgical scissors and treated with 0.1% pepsin solution (1.5 milliAnson units/mg; Serva, Germany) for 10 minutes at room temperature under magnetic stirring to maximise the release of germ cells from the seminiferous tubules. The cell suspension was fixed in 70% cold ethanol. Samples were then stored at -20°C prior to analysis. Fixed cells were treated with 0.5% pepsin solution for 10 minutes at room temperature under magnetic stirring and were stained with a solution containing 50 μg/ml propidium iodide (Sigma-Aldrich), and 40 μg/ml RNase (Sigma-Aldrich), in PBS. The propidium iodide-stained cells were analyzed in a FACSCalibur flow cytometer (Becton-Dickinson Immunocytometry, CA, USA). The fluorescent signals of propidium iodide-stained cells were recorded, and a cytogram of DNA content vs. cell count was used to identify cell populations on the basis of their DNA content. Typical DNA content fluorescence intensity distribution histograms from adult mouse testicular cells are characterized by distinct peaks comprising cells with different DNA content. The first region (1C) includes post-meiotic haploid spermatids. Diploid G1-phase spermatogonia, and secondary spermatocytes are recorded in the second peak (2C), together with terminally differentiated testis somatic cells. The third peak (4C) includes some G2/M spermatogonia but, mostly, primary spermatocytes. Actively DNA-synthesizing S-phase cells are located in the region between the second and the third peaks.

A total of 10,000 events were recorded for each histogram. The relative frequencies of testicular cell types were calculated using the Cell Quest software.

### Statistical analysis

Survival differences between the different groups were analysed through Mantel’s procedure of log-rank test. Multiple linear regressions were performed to assess if weight and micronucleus frequencies were dependent upon sex, litter size and treatment. Since no differences (using the Student’s t-test) were detected between the two litters of the same group, a mean value relative to all the pups treated in the same way was calculated and used for inter-groups comparison. Welch’s t-test was used to compare the micronucleus frequencies of the different treatment groups. One-way ANOVA and Duncan’s test was used for post-hoc comparison of male germ cell parameters and comet assay data among the different treatment groups. The level of significance was established at p<0.05. All tests were performed using Statistica 10 (Statsoft., Inc., OK, USA).

## Results

### Birth and development

Table 1 shows litter size, weights at birth and at weaning, and survival at weaning of the four experimental groups. Neither magnetic fields nor X-rays seemed to affect litter size. Survival was not significantly different between the groups. Multiple regression analysis showed that the effects of sex and litter size on weight were greater than the effect of treatment.

**Table 1:** 

	Number of pups (two litters)	Sex ratio (M:F)	Weight at birth (means±SD)	Weight at weaning (means±SD)	Survival at weaning
**C**	27	0.93	1.52±0.09 g	9.99±0.81 g	88.9%
**E**	20	1.22	1.53±0.20 g	9.20±1.48 g	95%
**X**	25	1.50	1.22±0.10 g	8.15±1.45 g	92%
**XE**	31	0.82	1.34±0.10 g	7.60±1.25 g	80.6%

In agreement with literature data [36], appearance of physiological landmarks (pinna detachment, eye opening, fur development, and testes descent) was delayed in the X-rays treated compared to the control group (data not shown). No differences were recorded between groups C and E and between groups X and XE.

### Micronucleus test on erythrocytes

A multiple regression analysis showed that micronucleus frequencies were not significantly associated either with sex or litter size. No significant differences were detected between the mean micronucleus frequencies of the two litters of the same treatment group.

**Fig 1:**
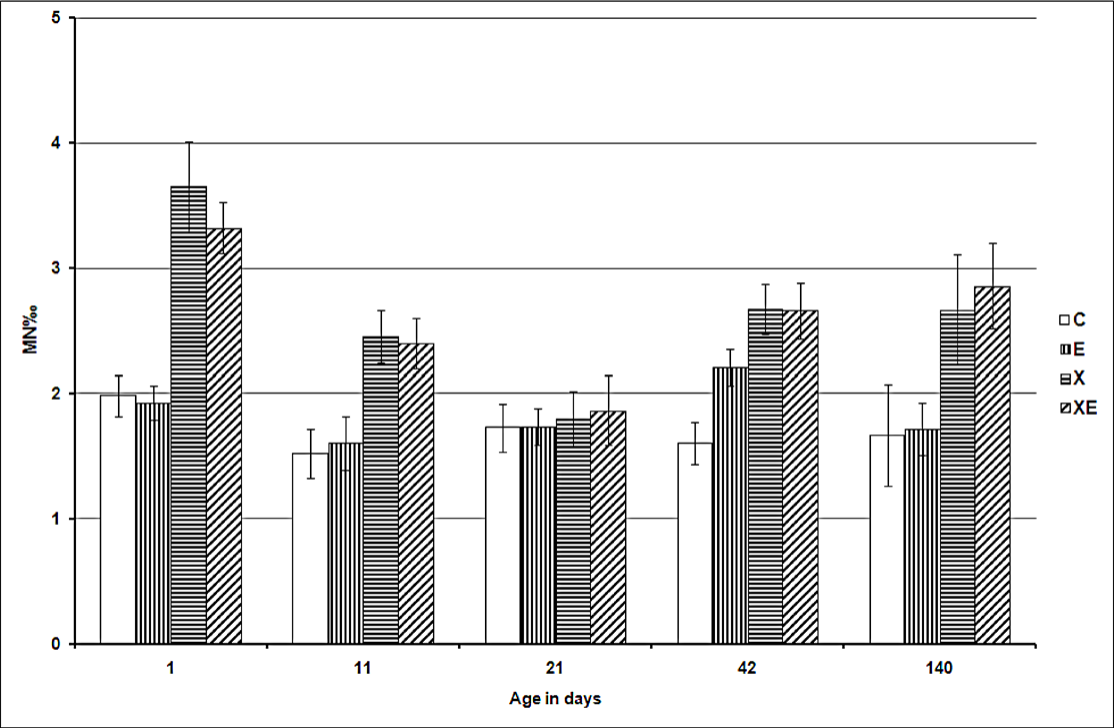
Micronucleus frequencies in peripheral blood erythrocytes. The number of animals in each group is reported inside the histogram columns. Bars represent standard error. Significance compared to C: * *p*<0.05; ** *p*<0.01; *** *p*<0.001.

Fig 1 shows the mean frequencies of micronucleated erythrocytes in blood sampled at different times in the four groups. At birth, the differences of micronucleus (MN) frequencies were statistically significant between groups C and X and between groups C and XE (*p*<0.001, both). Similarly, at day 11, the differences of MN frequencies between groups C and X and between groups C and XE were also statistically significant (*p* = 0.002 and *p* = 0.0057, respectively). At day 21, the mean frequencies of MN in C, E, X, and XE groups did not differ significantly. At day 42, the mean frequencies of MN in E, X and XE were significantly different compared to C (respectively *p* = 0.008, *p* = 0.02 and *p*<0.001). At day 140 the group XE showed a MN frequency statistically higher than that of the control group (*p* = 0.0048). Group X was also higher than C, but the differences was not signinficant due to a greater standard error (at that time the number of animals was greatly reduced). It should be also noted that in group X (as well as Group XE), the frequency of micronucleated erythrocytes were not different between day 42 and 140. The difference between groups X and XE was never statistically significant.

### Effects on the male reproductive system

**Fig 2:**
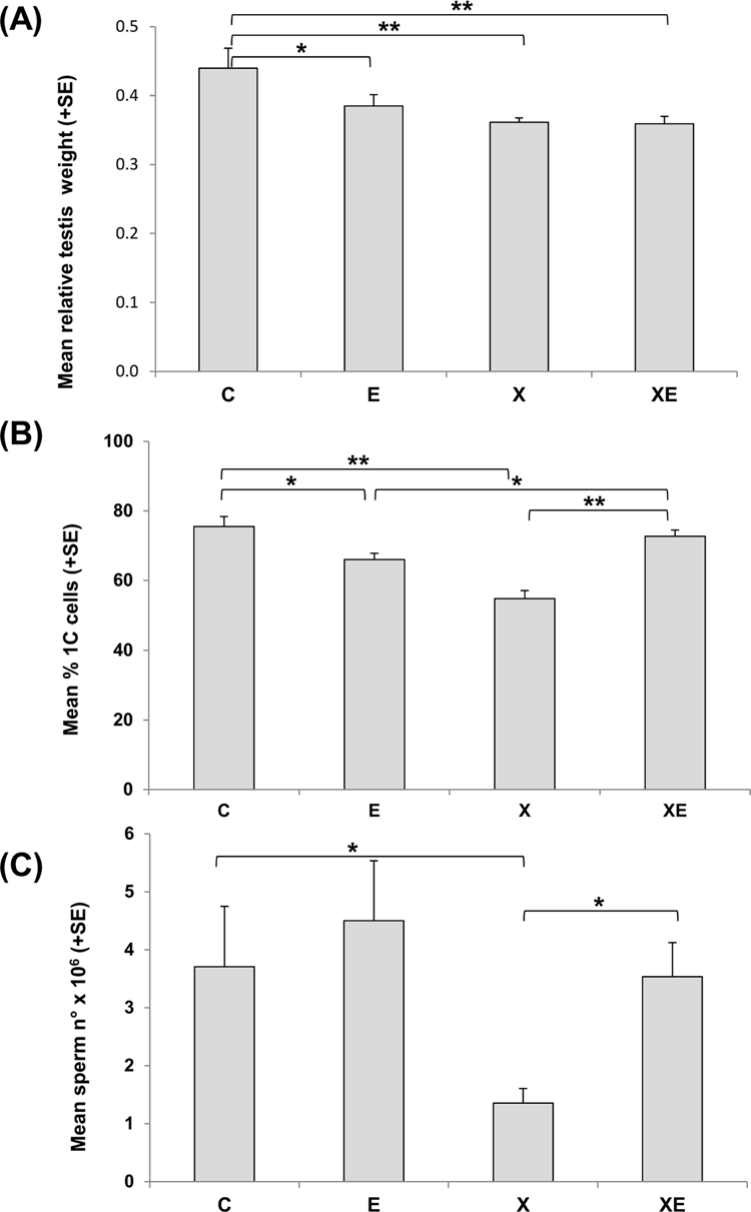
(A) Relative testicular weights (testis weight/body weight * 100); (B) percentages of 1C post meiotic cells as evaluated by DNA content flow cytometric analysis of whole testis cells; (C) sperm number in cauda epididymis. Columns represent the mean values of 7 controls (C), 11 ELF-MF exposed (E), 14 X-rays exposed (X), or 11 animals exposed to combined treatments (XE). Asterisks mark statistically different values between groups, *: *p*<0.05; **: *p*<0.005.

For the analyses of the effects on the male reproductive system, all the animals belonging to the 4 experimental groups were sacrificed 42 days after birth. The impact of the exposure(s) was evaluated by the capacity of the system to produce terminally differentiated germ cells and the level of damage in their DNA. Prenatal irradiation with a single X-rays dose at the time of early gonad differentiation induced a significant decrease of testis weight suggestive of a toxic effect (Fig 2A). ELF-MF exposure from 11.5 days post-conception to 21 days after birth induced a slight decrease of relative testis weight mainly due to a small increase of body weight. After combined exposure to ionizing radiation and ELF-MF, the level of testis weight reduction was comparable to that induced by X-rays exposure.

By flow cytometric analyses of testicular cells, the relative percentages of 1C, 2C, S-phase and 4C cells were calculated. A significant 28% reduction of postmeiotic 1C cells was observed after X-rays exposure, and a small but significant 13% reduction was detected also after ELF-MF exposure (Fig 2B). Surprisingly, after the combined exposure, the percentage of 1C cells was significantly higher than after the single treatments and equal to that of controls. The evidence of X-rays induced toxicity was magnified by the analysis of terminally differentiated cells in cauda epididymis where a 73% sperm count reduction was detected (Fig 2C). ELF-MF exposure did not induce a significant variation of sperm count with respect to controls. Consistently with the data on the relative percentages of testicular 1C cells, epididymal sperm count after combined exposure was significantly higher than after X-rays alone and not different from unexposed controls. Data on epididymis weight were consistent with those on sperm count (data not shown).

**Fig 3:**
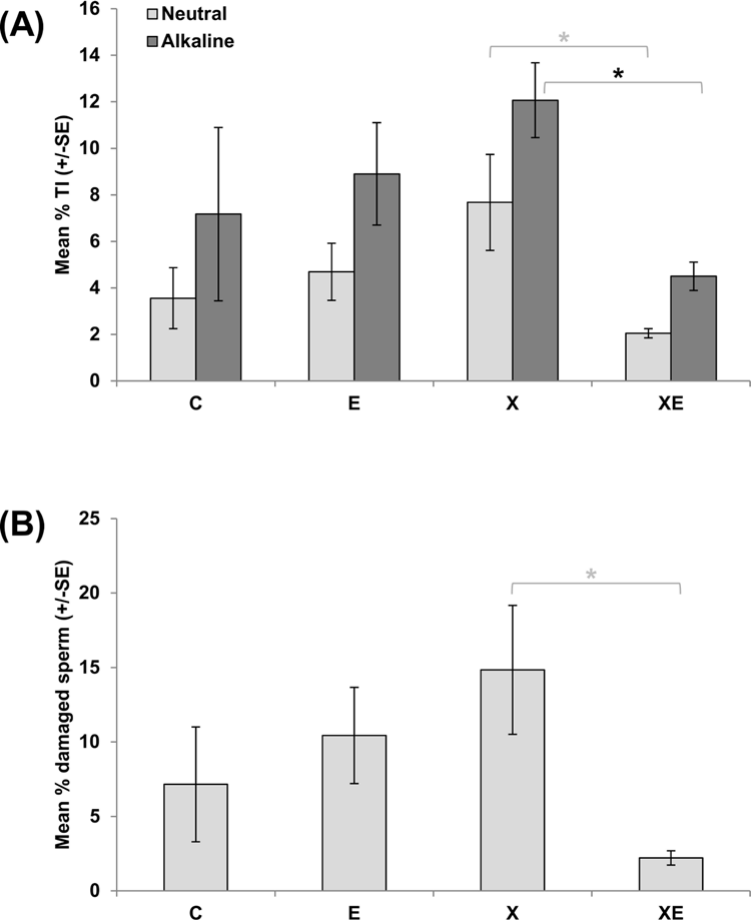
Comet assay in epididymal sperm. (A) Mean tail intensity values obtained with alkaline and neutral comet assay. (B) Percentage of sperm carrying clearly damaged DNA (with tail intensity values higher than 10%) after neutral assay. Columns represent the mean values of 4 controls (C), 5 ELF-MF exposed (E), 8 X-rays exposed (X), or 6 animals exposed to combined treatments (XE).

The effects of single and combined exposures to ELF-MF and X-rays were further evaluated by comet analysis in epididymal sperm. No effect of single treatment with ELF-MF or X-rays was shown under both neutral and alkaline conditions (Fig 3A). Also the combined exposure did not induce significant variation with respect to controls, but when the data were compared with those collected after X-rays alone, a statistically significant reduction of the mean tail intensity was detected. Under neutral conditions, an arbitrary tail intensity cut off point was established, above which sperm were considered clearly damaged. Consistently with the data on the mean tail intensity, the percentages of clearly damaged sperm were not significantly different from controls under any treatment conditions, but the percentage of clearly damaged sperm induced by the combined exposure was significantly less than that induced by X-rays alone (Fig 3B).

## Discussion

The main aim of this study was to employ a sensitive model to detect the genotoxic properties of ELF-MF, since–after decades of studies–proofs are still contradictory. Hence, we investigated the genotoxic effects of ELF-MF *in vivo* on somatic and germ cells after exposure during prenatal and neonatal developmental stages, known to be sensitive to environmental stress. In addition, genetic changes arising during this period of life might represent a co-factor at the origin of childhood cancer [37, 38]. We also aimed at investigating *in vivo* a possible influence of ELF-MF on the cell response to ionizing radiation. Chronic exposure to ELF-MF from day 12 p.c. until weaning had no teratogenic effect and did not affect survival, growth and development. X-irradiation at day 12 p.c. resulted in stunted pups and retarded development, in agreement with literature data [36]. No synergistic effects of ELF-MF and X-rays exposure on these parameters were observed.

Beside these developmental endpoints, we studied the genotoxic effects on the erythroid and male germ lines, using techniques validated in the respective tissues.

The results of the micronucleus test in peripheral blood seem to indicate that exposure to ELF-MF induced a slight genotoxic insult that became detectable only during a restricted expression time window, after the maximum exposure time (one month, including both a prenatal and a neonatal period). This result is different, but not in contradiction with the result obtained by Abramsson-Zetterberg and Grawé [9], who carried on the exposure during pregnancy, stopped it at birth and sampled blood from the prenatally exposed mice at the age of 35 days, finding no increase in MN frequencies. In fact, the differences in the duration and developmental windows of exposure might explain the different results.

The observed weak genotoxic effect of ELF-MF, only observed 42 days after birth, could be due to a perturbation of the cellular defence capacity against endogenous sources of DNA lesions.For the effect to be detectable both the exposure and expression times would be critical: a continuous exposure from mid-gestation until weaning, followed by a “latency” period, with a further haematopoietic system development seemed to be necessary. After 4 months the system appeared to have recovered its homeostatic capacity. Specific experiments should be conducted to identify the molecular mechanisms leading to genotoxic damage. Disturbance of the oxidative balance might be a candidate because many authors suggested that this mechanism is implicated in ELF-MF-induced biological effects [39,40].

Differently, exposure to X-rays seems to produce two distinct periods of detectable genotoxic effects. An elevated frequency of MN was observed at birth as a direct effect of X-irradiation. At the time when this happened (day 12 p.c.), the foetal liver was beginning erythropoiesis thanks to recently arrived hematopoietic stem cells [41] and micronucleated erythrocytes deriving from those progenitors/precursors were detected in circulating blood at birth. Consistently with the kinetics of prenatal erythropoiesis, the frequency of micronucleated erythrocytes observed in blood was one order of magnitude less than that detected in the foetal haematopoietic liver at the time of maximum expression (16–18 h) after a comparable radiation treatment [42]. As more time passed by, these micronucleated cells were further diluted in the bloodstream by erythrocytes formed from unirradiated precursors. In fact, MN frequency of the X group at day 11 is lower than at birth and reaches the control value at day 21. Beside this acute, early outcome, what it seems to be a delayed effect became detectable at day 42 and persisted until day 140. Finally, the data show that ELF-MF exposure did not modify the effect of X-rays at any tested time.

The effects on the male reproductive system were evaluated 42 days after birth when all germ cell sub-populations are present in the testis, the spermiogenesis has started and the first wave of spermatozoa is present in the epididymus. However, at this age the mouse germinal tissue is still in development as shown by a few comparative data: the testis weight was slightly lower than that of adults (0.11 vs 0.14 g respectively) and the number of spermatozoa was less than 15% of that found in adult mice.

Prenatal X-rays exposure at day 11.5 p.c. had an impact on testicular development as shown by the decrease of testis weight, of the relative frequency of post-meiotic cells (1C) and of the number of epididymal spermatozoa at 42 days after birth. These results are in agreement with the known radiosensitivity of germ and somatic cells in the foetal gonads [43].

ELF-MF exposure from 11.5 days post-conception to 21 days after birth induced a slight relative decrease of post-meiotic cells, which was not reflected in a decrease of spermatozoa. Studies on the effect of *in utero* exposure to ELF-MF on male reproductive system are few and results are controversial. A disorganization of spermatogenic epithelium was observed in adult rats as a result of oxidative stress induced by *in utero* exposure to ELF-MF, 3 mT, 4 h/day [28]. Testicular alteration was observed also in rats exposed from 13 days p.c. to 21 days after birth to ELF-MF 1 mT, 3 applications of 30 minutes per day [29]. An increase of weight in epididymus, prostate and seminal vesicles without differences in epididymal sperm count, was observed in adult rats exposed *in utero* to 15 Hz pulsed electromagnetic field from day 15 to day 20 of gestation [27]. On the other hand, *in utero* and neonatal exposure of rats to ELF-MF at field strengths up to 500 μT did not produce any alteration in the offspring spermatogenesis and fertility [26]. No reproductive or developmental toxic effects were shown in a multigeneration study in rats exposed 18.5 hours/day to ELF-MF at field strengths up to 1 mT [25].

To our knowledge, no studies have been conducted to assess the interaction of X-rays and ELF-MF pre-natal exposure in mammals. Pafkova and co-workers [44] reported that 10 mT of magnetic field modified the embryotoxic effect of ionizing radiation on chick embryos. Our results suggest that the continuous ELF-MF exposure from 11.5 day p.c., immediately after X-rays irradiation, to 21 days after birth, modulate the toxic effect of ionizing radiation as shown by the higher percentage of post-meiotic testicular cells and number of epididymal sperm in XE with respect to X experimental group. Our experimental protocol did not aim at studying the possible early mechanisms at the basis of this effect, but several hypotheses can be drawn: ELF-MF could influence DNA damage response stimulating DNA repair or inhibiting apoptosis; in addition, an impact of ELF-MF could be envisaged on proliferation of cells surviving the X-rays exposure, which could also influence the onset of puberty.

In our study we also aimed at assessing possible delayed effects on DNA of terminally differentiated spermatozoa. The induction of delayed effects in male germ cells has been supported by the observation of DNA damage in spermatozoa deriving from X-rays exposed adult spermatogonia [35,45–47]. In the present study a small, not significant increase of DNA damage was observed after *in utero* exposure to 1 Gy X-rays. It should be noted that the level of DNA damage in spermatozoa of untreated 42 days old mice was higher and more heterogeneous than in adult mice (laboratory historical data not shown). This result is in agreement with the characteristics of sperm populations produced by the first wave of spermatogenesis, which, in comparison with adult sperm show abnormalities in sperm head morphology and abnormal chromatin structure [48]. No effects were detected after continuous exposure to 50 Hz, 65 μT ELF-MF. Surprisingly, an evident reduction of DNA damage, homogeneous in all animals of the group, was observed when X-rays were followed by ELF-MF exposure. This effect, together with the observed increase of sperm number, suggests that ELF-MF is able to modulate male germ cell response to X-rays. This finding could be due to an influence of ELF-MF on early DNA damage response leading to a reduction of X-rays cell killing. Moreover, ELF-MF could influence post-natal germ cell proliferation/differentiation pathways, possibly anticipating the starting of spermatogenesis at puberty; this would result in a dilution of the first wave of naturally occurring abnormal spermatozoa with unaltered sperm deriving from a second wave of spermatogenesis. Although such an influence of ELF-MF on experimentally altered spermatogenesis has never been studied, the interaction of ELF-MF with damaged specific biological systems can modulate the proliferation/differentiation process such as the promotion of healing of bone fractures [4,49].

The intensity of the magnetic field we used was quite high; nonetheless, it is in the order of magnitude of some domestic exposure, for example in the case of holding a laptop computer on the womb. In the germinal tissue, we were able to detect a biological effect of ELF-MF particularly as modulation of the cell response to the toxic insult induced by a single X-rays dose. This result confirms the importance to investigate the effects of combined exposure to ELF-MF and other stressors because the modulation of a stress response may be as critical for the cell fate as the stress itself. Our data supported the hypothesis that the plasticity of developing tissues make them vulnerable to external stressors. Moreover, if ELF-MF may indeed interfere with proliferation/differentiation processes during early phases of life, it is important to investigate different tissues/organs because each one has its own specific differentiation pattern in a specific time window of development. This in turn points to the need of a deeper comprehension of the molecular mechanisms underlying ELF-MF biological effects.

## Supporting Information

S1 Data Individual values for the micronucleus test (Dataset1) and for the male reproductive system (Dataset2). (PDF)

## References

[1] Wertheimer N, Leeper E. Electrical wiring configurations and childhood cancer. Am J Epidemiol. 1979; 109(3): 273–284. 453167 10.1093/oxfordjournals.aje.a112681

[2] Teepen JC, van Dijck JA. Impact of high electromagnetic field levels on childhood leukemia incidence. Int J Cancer. 2012; 131(4): 769–778. 10.1002/ijc.27542 22437882

[3] Repacholi M. Concern that "EMF" magnetic fields from power lines cause cancer. Sci Total Environ. 2012; 426:454–458. 10.1016/j.scitotenv.2012.03.030 22534362

[4] IARC Working Group on the Evaluation of Carcinogenic Risks to Humans. Non-ionizing radiation, Part 1: static and extremely low-frequency (ELF) electric and magnetic fields. IARC Monogr Eval Carcinog Risks Hum. 2002; 80: 1–395. 12071196 PMC5098132

[5] World Health Organization. Environmental health criteria 238, Extremely Low Frequency Fields; 2007.

[6] Udroiu I, Giuliani L, Ieradi LA. Genotoxic properties of extremely low frequency electromagnetic fields In: Giuliani L, Soffritti M, editors. Non-thermal effects and mechanisms of interaction between electromagnetic fields and living matter. Fidenza (Italy): Mattioli 1885; 2010. p.123–134.

[7] Morita T, Asano N, Awogi T, Sasaki YF, Sato S, Shimada H, et al Evaluation of the rodent micronucleus assay in the screening of IARC carcinogens (groups 1, 2A and 2B) the summary report of the 6th collaborative study by CSGMT/JEMS MMS. Collaborative Study of the Micronucleus Group Test. Mammalian Mutagenicity Study Group. Mutat Res. 1997; 389(1): 3–122. 9062586 10.1016/s1383-5718(96)00070-8

[8] Svedenstal BM, Johanson KJ. Leukocytes and micronucleated erythrocytes in peripheral blood from mice exposed to 50-Hz or 20-kHz magnetic fields. Electro- Magnetobiol. 1998; 17: 127–143.

[9] Abramsson-Zetterberg L, Grawé J. Extended exposure of adult and fetal mice to 50 Hz magnetic field does not increase the incidence of micronuclei in erythrocytes. Bioelectromagnetics. 2001; 22(5): 351–357. 11424159 10.1002/bem.61

[10] Udroiu I, Cristaldi M, Ieradi LA, Bedini A, Giuliani L, Tanzarella C. Clastogenicity and aneuploidy in newborn and adult mice exposed to 50 Hz magnetic fields. Int J Radiat Biol. 2006; 82(8): 561–567. 16966183 10.1080/09553000600876660

[11] Udroiu I, Cristaldi M, Ieradi LA, Bedini A, Giuliani L. Genotoxic and hematotoxic damage induced by ELF magnetic fields. Eur J Oncol. 2008; 13: 239–244.

[12] Erdal N, Gürgül S, Celik A. Cytogenetic effects of extremely low frequency magnetic field on Wistar rat bone marrow. Mutat Res. 2007; 630(1–2): 69–77. 17452120 10.1016/j.mrgentox.2007.03.001

[13] Okudan N, Celik I, Salbacak A, Cicekcibasi AE, Buyukmumcu M, Gökbel H. Effects of long-term 50 Hz magnetic field exposure on the micronucleated polychromatic erythrocyte and blood lymphocyte frequency and argyrophilic nucleolar organizer regions in lymphocytes of mice. Neurol Endocrinol Lett. 2010; 31: 208–214. 20424591

[14] Alcaraz M, Olmos E, Alcaraz-Saura M, Achel DG, Castillo J. Effect of long-term 50 Hz magnetic field exposure on the micronucleated polychromatic erythrocytes of mice. Electromagn Biol Med. 2014; 33(1): 51–57. 10.3109/15368378.2013.783851 23781994

[15] Sanderson BJ, Clark AM. Micronuclei in adult and foetal mice exposed in vivo to heliotrine, urethane, monocrotaline and benzidine. Mutat Res. 1993; 285(1): 27–33. 7678129 10.1016/0027-5107(93)90048-k

[16] Udroiu I, Ieradi LA, Cristaldi M, Tanzarella C. Detection of clastogenic and aneugenic damage in newborn rats. Environ Mol Mutagen. 2006; 47(5): 320–324. 16538686 10.1002/em.20209

[17] Zúñiga-González G, Ramírez-Muñoz MP, Torres-Bugarín O, Pérez-Jiménez J, Ramos-Mora A, Zamora-Pérez A, Gallegos-Arreola MP, Sánchez-Corona J. Induction of micronuclei in the domestic cat (Felis domesticus) peripheral blood by colchicine and cytosine-arabinoside. Mutat Res. 1998; 413(2): 187–189. 9639702 10.1016/s1383-5718(97)00184-8

[18] Neri M, Ceppi M, Knudsen LE, Merlo DF, Barale R, Puntoni R, Bonassi S. Baseline micronuclei frequency in children: estimates from meta- and pooled analyses. Environ Health Perspect. 2005; 113(9): 1226–1229. 16140632 10.1289/ehp.7806 PMC1280406

[19] Paashuis-Lew YR, Heddle JA. Spontaneous mutation during fetal development and post-natal growth. Mutagenesis. 1998; 13(6): 613–617. 9862193 10.1093/mutage/13.6.613

[20] Li P, McLaughlin J, Infante-Rivard C. Maternal occupational exposure to extremely low frequency magnetic fields and the risk of brain cancer in the offspring. Cancer Causes Control. 2009; 20(6): 945–955. 10.1007/s10552-009-9311-5 19224378

[21] Vergouwen RP, Jacobs SG, Huiskamp R, Davids JA, de Rooij DG. Proliferative activity of gonocytes, Sertoli cells and interstitial cells during testicular development in mice. J Reprod Fertil. 1991; 93(1): 233–243. 1920294 10.1530/jrf.0.0930233

[22] Chernoff N, Rogers JM, Kavet R. A review of the literature on potential reproductive and developmental toxicity of electric and magnetic fields. Toxicology. 1992; 74(2–3): 91–126. 1519247 10.1016/0300-483x(92)90132-x

[23] Lee SK, Park S, Gimm YM, Kim YW. Extremely low frequency magnetic fields induce spermatogenic germ cell apoptosis: possible mechanism. Biomed Res Int. 2014; 2014:567183 10.1155/2014/567183 25025060 PMC4082851

[24] Duan W, Liu C, Wu H, Chen C, Zhang T, Gao P, Luo X, Yu Z, Zhou Z. Effects of exposure to extremely low frequency magnetic fields on spermatogenesis in adult rats. Bioelectromagnetics. 2014; 35(1): 58–69. 10.1002/bem.21816 24122970

[25] Ryan BM, Symanski RR, Pomeranz LE, Johnson TR, Gauger JR, McCormick DL. Multigeneration reproductive toxicity assessment of 60-Hz magnetic fields using a continuous breeding protocol in rats. Teratology. 1999; 59(3): 156–162. 10194806 10.1002/(SICI)1096-9926(199903)59:3<156::AID-TERA7>3.0.CO;2-B

[26] Chung MK, Lee SJ, Kim YB, Park SC, Shin DH, Kim SH, Kim JC. Evaluation of spermatogenesis and fertility in F1 male rats after in utero and neonatal exposure to extremely low frequency electromagnetic fields. Asian J Androl. 2005; 7(2): 189–194. 15897976 10.1111/j.1745-7262.2005.00007.x

[27] McGivern RF, Sokol RZ, Adey WR. Prenatal exposure to a low-frequency electromagnetic field demasculinizes adult scent marking behavior and increases accessory sex organ weights in rats. Teratology. 1990; 41(1): 1–8. 2106174 10.1002/tera.1420410102

[28] Gharamaleki H, Parivar K, Soleimani Rad J, Roshangar L, Shariati M. Effects of electromagnetic field exposure during the prenatal period on biomarkers of oxidative stress and pathology of testis and testosterone level of adult rats in F1 generation. Acta Endo. (Buc.) 2014; 10: 577–587.

[29] Tenorio BM, Jimenez GC, Morais RN, Torres SM, Albuquerque Nogueira R, Silva Junior VA. Testicular development evaluation in rats exposed to 60 Hz and 1 mT electromagnetic field. J Appl Toxicol. 2011; 31(3): 223–230. 10.1002/jat.1584 20936650

[30] Hintenlang DE. Synergistic effects of ionizing radiation and 60 Hz magnetic fields. Bioelectromagnetics. 1993; 14(6): 545–551. 8297398 10.1002/bem.2250140606

[31] Miyakoshi J, Yoshida M, Shibuya K, Hiraoka M. Exposure to strong magnetic fields at power frequency potentiates X-ray-induced DNA strand breaks. J Radiat Res. 2000; 41(3): 293–302. 11210830 10.1269/jrr.41.293

[32] Yoon HE, Lee JS, Myung SH, Lee YS. Increased γ-H2AX by exposure to a 60-Hz magnetic fields combined with ionizing radiation, but not hydrogen peroxide, in non-tumorigenic human cell lines. Int J Radiat Biol. 2014; 90(4): 291–298. 10.3109/09553002.2014.887866 24467330

[33] Karotki AV, Baverstock K. What mechanisms/processes underlie radiation-induced genomic instability? Cell Mol Life Sci. 2012; 69(20):3351–60. 10.1007/s00018-012-1148-5 22955377 PMC11115179

[34] Xi W, Stuchly MA, Gandji OP. Induced electric currents in models of man and rodents from 60 Hz magnetic fields. IEEE TransBiomed Eng. 1994; 41: 1018–1023. 10.1109/10.335839 8001990

[35] Cordelli E, Fresegna AM, Leter G, Eleuteri P, Spanò M, Villani P. Evaluation of DNA damage in different stages of mouse spermatogenesis after testicular X irradiation. Radiat Res. 2003; 160(4): 443–451. 12968930 10.1667/rr3053

[36] Brent RL. Radiation teratogenesis. Teratology. 1980; 21(3): 281–298. 7006137 10.1002/tera.1420210304

[37] Marshall GM, Carter DR, Cheung BB, Liu T, Mateos MK, Meyerowitz JG, Weiss WA. The prenatal origins of cancer. Nat Rev Cancer. 2014; 14(4): 277–289. 10.1038/nrc3679 24599217 PMC4041218

[38] Kim AS, Eastmond DA, Preston RJ. Childhood acute lymphocytic leukemia and perspectives on risk assessment of early-life stage exposures. Mutat Res. 2006; 613(2–3): 138–160. 17049456 10.1016/j.mrrev.2006.09.001

[39] Luukkonen J, Liimatainen A, Juutilainen J, Naarala J. Induction of genomic instability, oxidative processes, and mitochondrial activity by 50Hz magnetic fields in human SH-SY5Y neuroblastoma cells. Mutat Res. 2014;760:33–41. 10.1016/j.mrfmmm.2013.12.002 24374227

[40] Mattsson MO, Simkó M. Grouping of experimental conditions as an approach to evaluate effects of extremely low-frequency magnetic fields on oxidative response in in vitro studies. Front Public Health. 2014;2:132 10.3389/fpubh.2014.00132 25229055 PMC4151017

[41] Ema H, Nakauchi H. Expansion of hematopoietic stem cells in the developing liver of a mouse embryo. Blood. 2000; 95(7): 2284–2288. 10733497

[42] Cole RJ, Taylor N, Cole J, Arlett CF. Short-term tests for transplacentally active carcinogens. I. Micronucleus formation in fetal and maternal mouse erythroblasts. Mutat Res. 1981; 80(1): 141–157. 7207479 10.1016/0027-5107(81)90184-6

[43] Vergouwen RP, Huiskamp R, Bas RJ, Roepers-Gajadien HL, Davids JA, de Rooij DG. Radiosensitivity of testicular cells in the fetal mouse. Radiat Res. 1995; 141(1): 66–73. 7997516

[44] Pafkova H, Jerabek J. Interaction of MF 50 Hz, 10 mT with high dose of X-rays: evaluation of embryotoxicity in chick embryos. Rev Environ Health. 1994; 10(3–4): 235–241. 7724884 10.1515/reveh.1994.10.3-4.235

[45] Haines GA, Hendry JH, Daniel CP, Morris ID. Increased levels of comet-detected spermatozoa DNA damage following in vivo isotopic- or X-irradiation of spermatogonia. Mutat Res. 2001; 495(1–2): 21–32. 11448639 10.1016/s1383-5718(01)00181-4

[46] Haines GA, Hendry JH, Daniel CP, Morris ID. Germ cell and dose-dependent DNA damage measured by the comet assay in murine spermatozoaa after testicular X-irradiation. Biol Reprod. 2002; 67(3): 854–861. 12193394 10.1095/biolreprod.102.004382

[47] Cordelli E, Eleuteri P, Grollino MG, Benassi B, Blandino G, Bartoleschi C, Pardini MC, Di Caprio EV, Spanò M, Pacchierotti F, Villani P. Direct and delayed X-ray-induced DNA damage in male mouse germ cells. Environ Mol Mutagen. 2012; 53(6): 429–439. 10.1002/em.21703 22730201

[48] Janca FC, Jost LK, Evenson DP. Mouse testicular and sperm cell development characterized from birth to adulthood by dual parameter flow cytometry. Biol Reprod. 1986; 34(4): 613–623. 3708046 10.1095/biolreprod34.4.613

[49] Zhang X, Zhang J, Qu X, Wen J. Effects of different extremely low-frequency electromagnetic fields on osteoblasts. Electromagn Biol Med. 2007; 26(3): 167–177. 17886004 10.1080/15368370701580756

