# Supplementary material for: Genotoxicity Induced by Foetal and Infant Exposure to Magnetic Fields and Modulation of Ionising Radiation Effects
Source: PLoS One. 2015 Nov 11;10(11):e0142259. doi: 10.1371/journal.pone.0142259 (PMC4641635; doi:10.1371/journal.pone.0142259)
Supplement: S1 Data — (PDF) [file pone.0142259.s001.pdf]

DATASET1: MICRONUCLEUS TEST ON PERIPHERAL BLOOD

Values represent micronucleated erythrocytes per 1000 erythrocytes

Birth

	CONTROL	ELF	XRAY	ELF+XRAY
	3	2	4,5	5
	1,5	3	3,5	2,5
	1,5	2	5	4,5
	1	0,5	1,5	2
	2,5	1,5	9	2,5
	1,5	1,5	3	4
	3,5	1,5	3	4
	1,5	1,5	4	2
	1,5	3	3	3
	3,5	2	3	1,5
	2,5	2	3	4,5
	3	2	6	3
	2,5	1,5	3	3
	2	2	3,5	4
	0,5	2	1	1,5
	2,5	2,5	2	4
	1	1,5	6	3
	2	2	3,5	3,5
	1,5	3	3	5
	3	1,5	5	4,5
	1		4	2,5
	3		2,5	1,5
	2,5		2	4
	1,5			3,5
	1,5			5
	1,5			3
	1			2
				5
				4
				2
				3,5
**mean**	**1,9815**	**1,925**	**3,6522**	**3,3226**
std.dev.	0,837596	0,612909	1,728339	1,122114

Day 11

	CONTROL	ELF	XRAY	ELF+XRAY
	2,5	1	3,5	2
	1	2	3,5	3,5
	1,5	2	1,5	1
	3	3	1	2
	0,5	1	2	2
	0	0,5	3,5	2
	0,5	2	2,5	3
	1,5	1,5	3,5	2,5
	1	1	3,5	3
	2	0,5	2	2,5
	0,5	2	2	3,5
	2,5	2	3,5	2
	1,5	3	0	1
	1,5	3	2,5	3
	1	1	1	3
	1	3	2	2
	2,5	0	3	2
	0	1	2,5	1,5
	2,5	1	3	3
	1,5		2	2,5
	3		4	3,5
	2		2,5	1,5
	3		2	3,5
	0,5			3
				2
**mean**	**1,5208**	**1,6053**	**2,4565**	**2,42**
std.dev.	0,94959	0,936586	0,999011	0,759386

Day 21

	CONTROL	ELF	XRAY	ELF+XRAY
	1	2	3	5
	0	1	2	2
	2	1	4	1,5
	1	2	2	1,5
	3	1,5	1	1
	0,5	2,5	2	2
	1	1	2	2
	2	2	3	1,5
	2	2	2	1
	1	1	1	0,5
	3	2	4	1
	2	2	1	3
	2	2	1	2
	1	2	2	2
	2	1	2	2
	2	2	1	1
	0,5	2	1	2
	2	1	0,5	1
	1	3	2	1,5
	1		2	2,5
	3,5		1	2
	3		0	2,5
	3		2	1,5
	2			3,5
				1
**mean**	**1,7292**	**1,7368**	**1,8043**	**1,86**
std.dev.	0,932262	0,586146	1,008364	0,95219

Day 42

	CONTROL	ELF	XRAY	ELF+XRAY
	3	3	3	3
	2	2	3	2
	2	3	1	2
	1	2	4	2
	2	1	3	1
	2	3	2	2
	2	2	3	1
	3	2	3	3,5
	2	3	2	2
	0	3	5	4
	1	1	1	3
	1	2	2	1
	1	2	2	2
	1	2	2	5
	2	3	3	2
	1	2	3	3
	1	2	2	2
	1	2	3	4
	2	2	3	3
	2		2	4
			2	3
			4,5	2
			3	2
				5
				3
**mean**	**1,6**	**2,2105**	**2,6739**	**2,66**
std.dev.	0,753937	0,630604	0,972446	1,124722

Day 140

	CONTROL	ELF	XRAY	ELF+XRAY
	2	2	1	4
	3	2	4	2
	0	2	2	3
	2	1	2	4
	1	1	1	2
	2	2	3	2
		2	3	3
		2	3	
			5	
**mean**	**1,6667**	**1,75**	**2,6667**	**2,8571**
std.dev.	1,032796	0,46291	1,322876	0,899735

DATASET2: SUMMARY OF RESULTS (SINGLE ANIMAL DATA AND MEANS OF EXPERIMENTAL GROUPS) RELATIVE TO MALE REPRODUCTIVE SYSTEM

	Relative testicular weights	Flow cytometry	Sperm number x 10^6	Comet assay		
				alkaline	neutral	
		% 1C cells		% TI	% TI	% dameged cells
Control						
	0,44	83,01	5,00	3,56	0,52	0,00
	0,56	82,03	7,40	2,23	3,04	5,83
	0,53	62,59	0,60			
	0,36	67,99	1,50	18,24	6,89	18,09
	0,40	79,94	3,90	4,65	3,78	4,72
	0,40	79,13	0,95			
	0,39	73,77	6,60			
**mean**	**0,44**	**75,49**	**3,71**	**7,17**	**3,56**	**7,16**
ELF						
	0,38	71,36	3,20			
	0,36	65,29	2,20			
	0,38	69,73	5,60			
	0,34	68,39	2,50	6,13	2,65	7,02
	0,35	50,63	2,00	16,57	7,53	19,42
	0,36	61,91	2,60	9,94	7,37	16,35
	0,40	71,21	12,80			
	0,33	69,75	8,80			
	0,37	67,22	4,20			
	0,45	62,94	1,60	8,35	4,47	7,55
	0,52	67,99	4,00	3,51	1,44	1,85
**mean**	**0,39**	**66,04**	**4,50**	**8,90**	**4,69**	**10,44**
X ray						
	0,36	48,93	0,75	16,48	15,86	32,17
	0,36	49,23	1,10	13,88	9,08	17,92
	0,35	64,20	3,80	8,38	2,66	2,78
	0,36	40,63	1,30	20,50	16,97	34,00
	0,31	56,64	2,16			
	0,36	49,13	0,58			
	0,34	35,96	0,58			
	0,35	58,95	2,25			
	0,36	60,09	0,60	8,48	4,04	7,27
	0,38	60,50	1,25	7,77	6,85	13,33
	0,37	64,58	2,25	11,31	3,10	5,61
	0,37	59,18	0,60	9,73	2,88	5,66
	0,37	60,92	0,92			
	0,42	57,55	0,83			
**mean**	**0,36**	**54,75**	**1,36**	**12,07**	**7,68**	**14,84**
ELF+Xray						
	0,35	74,34	0,75			
	0,34	69,31	1,13			
	0,37	76,82	5,13			
	0,32	70,07	1,38	3,75	2,05	3,85
	0,32	72,19	7,13	4,35	2,65	1,89
	0,43	69,87	3,38	5,14	2,53	2,83
	0,37	67,69	3,70			
	0,36	71,07	4,80			
	0,35	87,35	4,20	4,55	1,97	0,91
	0,33	68,24	4,80	2,35	1,69	0,92
	0,40	72,69	2,50	6,87	1,40	2,86
**mean**	**25,89**	**0,36**	**3,53**	**4,50**	**2,05**	**2,21**
